# Incentives and rewards: what do adult populations truly want? Insights from the physical activity loyalty (PAL) scheme

**DOI:** 10.1186/1745-6215-16-S2-P108

**Published:** 2015-11-16

**Authors:** Helen McAneney, Ruth Hunter, Frank Kee, Mike Clarke

**Affiliations:** 1 Northern Ireland Network for Trials Methodology Research, Centre for Public Health, Queen's University Belfast, Belfast, UK; 2 Centre for Public Health, Queen's University Belfast, Belfast, UK; 3 Centre of Excellence for Public Health, Belfast, UK

## Background

Trials depend on good recruitment and retention, but efforts to improve these have had varying success. This may be due to inadequate understanding of what participants would value in return for taking part. An opportunity arose in one trial to investigate the incentives that might help recruit and retain participants to another.

## Aim

To determine what adults value as an incentive for involvement in a trial.

## Methods

In the PAL Scheme, employees used a ‘loyalty card’ to monitor their physical activity over 12 weeks. The incentive group (n=199) collected points and received rewards for physical activity (1 minute = 1 point, max: 30 pts/day). A comparator group (n=207) self-monitored their physical activity only. Points could be redeemed as retail vouchers. 17 different incentives were available, from 75 pts (£2.50, a sandwich) to 1800 pts (£60, 1 month gym membership).

## Results

148 of the 199 intervention participants used their card at least once, earning a mean of 374 pts. 121 earned sufficient to collect a reward and 76 redeemed points for vouchers but only 48 exchanged the vouchers for rewards. The most popular reward was not that of highest monetary value: two cinema tickets (300 pts, £10).

## Conclusions

The value that participants place on a reward might be more important than its monetary value. Some might appreciate receiving the voucher, without spending it. In choosing incentives to boost trial participation, it may help to allow people to choose from a variety of rewards, rather than reimbursing in money.

